# Association of COVID-19-related perceptions and experiences with depression and anxiety in Ugandan caregivers of young children with malaria and iron deficiency: A cross-sectional study

**DOI:** 10.1371/journal.pone.0314409

**Published:** 2024-12-10

**Authors:** Saeun Park, Paul Bangirana, Ezekiel Mupere, Reagan I. Baluku, Erika S. Helgeson, Sarah E. Cusick

**Affiliations:** 1 Division of Epidemiology and Community Health, University of Minnesota School of Public Health, Minneapolis, MN, United States of America; 2 Department of Psychiatry, Makerere University, Kampala, Uganda; 3 Global Health Uganda, Kampala, Uganda; 4 Department of Pediatrics and Child Health School of Medicine College of Health Sciences, Makerere University, Kampala, Uganda; 5 Division of Biostatistics and Health Data Science, University of Minnesota School of Public Health, Minneapolis, MN, United States of America; 6 Department of Pediatrics, University of Minnesota School of Medicine, Minneapolis, MN, United States of America; Jordan University of Science and Technology, JORDAN

## Abstract

**Background:**

Caregivers of young children may have been particularly vulnerable to mental health challenges during the COVID-19 pandemic due to its negative impacts on their housing, finances, and childcare demands. This study explored the associations between COVID-19-related experiences and symptoms of depression and anxiety among Ugandan caregivers.

**Methods:**

This cross-sectional study included 100 Ugandan caregivers of young children aged 6–59 months with uncomplicated malaria and iron deficiency (N = 85) and without malaria or anemia (N = 15) who were enrolled in the Optimizing Iron Status in Malaria-Endemic Areas (OptiM) study. Sociodemographic data and COVID-19 experiences were collected using an internally developed survey and symptoms of depression and anxiety were measured using the Hopkins Symptom Checklist (HSCL-25) and the Center for Epidemiologic Studies Depression (CESD-20) scale. Multiple linear regression models were used to assess the associations between COVID-19 survey scores with HSCL-25 or CESD-20 scores.

**Results:**

Nearly half of caregivers reported clinically meaningful symptoms of depression (46%) and/or anxiety (49%). Caregivers had more severe symptoms of depression and/or anxiety if they experienced greater changes in living situations or decreases in physical activity (CESD-20: β = 3.35, 95% CI [1.00, 5.70], p = .01), food insecurity (HSCL-25: β = 3.25, 95% CI [0.41, 6.10], p = .03, CESD-25: β = 3.09, 95% CI [0.79, 5.39], p = .01), and domestic violence (HSCL-25: β = 3.82, 95% CI [0.94, 6.70], p = .01) during COVID-19. These associations did not vary depending on whether the caregivers had children with malaria.

**Conclusions:**

Negative COVID-19 experiences were significantly associated with more severe depression and anxiety in Ugandan caregivers, regardless of their children’s malaria status. Urgent attention and action are needed to support the mental well-being of this vulnerable population. Further prospective studies should investigate the long-term impact of COVID-19 on caregivers and their children.

## Introduction

Since late 2019, Covid-19 has rapidly spread worldwide, adversely affecting many aspects of people’s lives, including their mental health [1]. The constant risk of contracting a disease without knowing when life would return to normal has itself been reported to have cause fear and stress during the pandemic [2]. Additionally, lockdowns and other social distancing measures imposed in response to COVID-19 led to reduced physical activity and changes in daily routines which led many to be lonely, anxious, and depressed [3]. Societal burdens imposed by the economic downturns, including unemployment and financial and food insecurity, have also adversely affected the mental well-being of people [4]. In particular, primary caregivers of young children carried a heavy burden [5, 6].

Uganda is an important country to investigate the mental health impact of COVID-19, considering its strict lockdown measures during the pandemic. Uganda implemented the most stringent lockdown measures in Africa to prevent the spread of the virus [7]. The Ugandan government adopted two "total lockdowns," enforcing closed borders, early curfews, closure of schools, prohibition of public gatherings, and the restriction of public transportation, including taxis and private cars, from March to June 2020 and June to July 2021 [8]. Although some measures were eased outside the total lockdown periods, some measures including school closures and nightly curfews, were put in place for more than 20 months since their first implementation and were not lifted until the end of January 2022 [9]. All these measures exacerbated existing societal and financial problems in Uganda, including unemployment and food insecurity, without a proper financial or mental support system [10]. Furthermore, during the lockdowns, security officers employed stringent measures to enforce the lockdown measures, which at times turned violent [11, 12]. This resulted in many Ugandans being directly or indirectly exposed to violent events, which may have played a critical role in developing trauma and mental stress [12]. All these social insecurities and stressors caused by the lockdowns are risk factors for mental health problems.

While previous studies in Uganda have reported increased levels of depression and anxiety among university students, low-income earners, and adults diagnosed with COVID-19 [13–15], no study has examined how societal problems and insecurities caused by COVID-19 affected the mental health of caregivers of young children in Uganda. As reported in other sub-Saharan countries that enforced strict lockdowns [16, 17], Ugandan caregivers are highly likely to experience stress coming from increased childcare needs without sufficient support from their family and community due to social distancing. Additionally, increased poverty and food insecurity caused by strict lockdowns may have added to their mental health burden [10]. Additionally, mothers who were pregnant or caregivers of children with illnesses may have been vulnerable to developing mental illness due to limited healthcare access during the lockdowns [7]. An increase in mental health problems among mothers can have devastating effects on themselves and their children [18]. In Uganda, like many other low- and middle-income countries, mental health care is severely under-resourced due to limited budget allocated to health care and other disease burden (e.g., malaria and HIV) [19]. Therefore, common mental health problems like depression and anxiety often remain undetected and untreated [18].

This is alarming because poor mental health among caregivers can result in altered childcare practices such as inadequate care for the nutrition and health of children and reduced responsive caregiving which, in turn, can lead to altered child development [20]. Disruptions in early childhood development can lead to adverse long-term and intergenerational consequences [21]. Altered child development is known to reduce the opportunity of achieving productive and healthy life as adults [22]. Despite these acknowledged risks, no study has investigated the adverse impact of COVID-19 on caregiver mental health in Uganda.

The objective of the present study was to explore whether COVID-19-related experiences were related to depression and anxiety among caregivers of young children. We hypothesized that more negative COVID-19-related perceptions and experiences would be associated with more severe symptoms of depression and anxiety among the caregivers. We also examined whether the association between COVID-19 experiences and mental health status varied depending on whether caregivers were caring for children with malaria or iron deficiency, based on the possibility that caring for ill children may have made caregivers more mentally vulnerable to COVID-19-related stressors. Although there is no direct evidence on the mental vulnerability of caregivers of children with acute illnesses such as malaria, previous studies have reported elevated levels of stress and depression among caregivers of children with chronic illnesses or disabilities [23, 24]. Findings from the current study can contribute to a better understanding of the mental health risks among caregivers of young children in Uganda and help plan for preventive measures or support for the mental well-being of the vulnerable population during unexpected pandemic situations like COVID-19.

## Methods

### Study population

**Fig 1:**
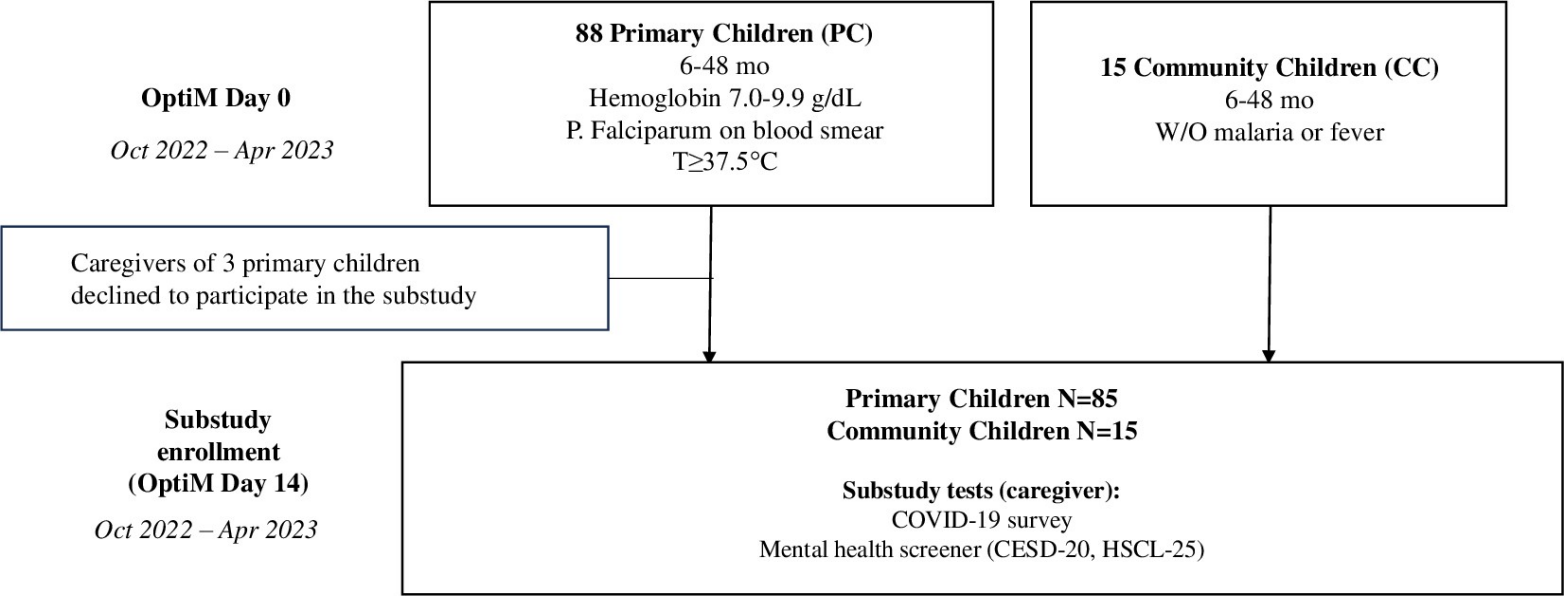
Timeline for study enrollment.

Between October 2022 and April 2023, we recruited 100 primary caregivers and their children who were enrolled in the Optimizing Iron Status in Malaria-Endemic Areas (OptiM, ClinicalTrials.gov: NCT03897673) study. The OptiM study began enrolling in October 2019 and is an ongoing, longitudinal, randomized, placebo-controlled clinical trial of iron supplementation and neurodevelopment in children with malaria and iron deficiency that is based at Makerere University/Mulago Hospital in Kampala and at Jinja Regional Referral Hospital in Jinja, Uganda. The primary caregivers of each child enrolled in the larger study were approached by the OptiM study medical officer or nurse 14 days after enrollment (on the day of baseline neuropsychological testing) to ask if they were interested in participating in the COVID sub-study (**Fig 1**).

The OptiM study enrolled children aged 6 to 48 months with malaria as a group of interest (primary children) and children of same age and from the same village or household as primary children as a control group (community children). To be eligible to participate in the OptiM study, primary children had to meet the following criteria: 1) Age 6–48 months at enrollment; 2) Hemoglobin 7.0–9.9 g/dL; 3) Zinc protoporphyrin (ZPP, a marker of iron deficiency) ≥ 80 μmol/mol heme; 4) *P*. *falciparum* positive by Giemsa smear or Rapid Diagnostic Test (RDT) positive; 5) Temperature ≥ 37.5°C or history of fever in past 24 hours. Inclusion criteria for community children were: 1) Living in the same neighborhood, extended household, or nearby neighborhood as a primary child; 2) Within one year of age of the primary child; 3) Hemoglobin ≥ 10.0 g/dL.

Exclusion criteria for primary children in OptiM: 1) Severe malaria, including severe anemia, prostration, cerebral malaria, repeated seizures or symptoms like persistent vomiting, high temperature (>39.5°C), or tea-colored urine, based on the criteria outlined in the *Management of Severe Malaria* by the World Health Organization [25]; 2) Severe malnutrition, evidenced by severe wasting or bilateral pitting edema; 3) Known sickle cell disease; 4) Acute hemorrhage; 5) Known cancer or leukemia; and 6) Caregiver does not understand English, Luganda, or Lusoga. Exclusion criteria for community children in OptiM: 1) Clinical malaria infection or any active illness within the past four weeks requiring medical care; 2) Chronic illness requiring medical care; 3) Major medical abnormalities on screening history or physical exam, including measured temperature ≥ 37.5°C; 4) Known developmental delay or neurologic disorder; 5) Prior history of coma; 6) Caregiver does not understand English, Luganda, or Lusoga; and 7) Other severe illness such as pneumonia or cardiac failure.

Caregivers of the OptiM children had to satisfy the following additional criteria to be enrolled in the COVID substudy: 1) they should be primary caregiver of a child enrolled in the OptiM study; and 2) at least 18 years of age. We excluded caregivers who were not mentally or physically incapable of participating in the survey and assessments. If a caregiver showed interest in participating in the sub-study, they underwent the written informed consent process and were enrolled in the COVID sub-study on the OptiM study Day 14 visit date (child neurobehavioral testing day). On the day of substudy enrollment, caregiver’s hemoglobin level was measured by finger-prick sample at enrollment using HemoCue (HemoCue AB, Angelhom Sweden) at the same study hospital, after which caregiver assessments, including baseline COVID-19 survey and mental health screener, were conducted. As described in **Fig 1**, caregivers of 85 children with malaria and iron deficiency (primary children) and 15 children without malaria or anemia (community children) were enrolled in our study. The higher number of children with malaria reflects the fact that these children were the primary group of interest in the parent iron supplementation study.

### Measures

#### COVID-19 survey

We developed a survey to collect information on caregivers’ experiences and perceptions towards COVID-19 based on existing COVID-19 surveys such as the COVID Experiences (COVEX) questionnaire and the Performance Monitoring for Action (PMA) COVID-19 survey [26, 27]. The survey we developed was intended to capture a wide range of caregiver’s experiences under COVID-19 that potentially had negative impacts on mental health. The survey included questions related to following COVID-19 topics: 1) general experience about infection status, testing, and vaccination history; 2) economic impact on job loss and food security; and 3) concern and perceived risk of infection. The survey also included questions related to sociodemographic information such as gender, age, marital status, and education level.

To ensure the relevance and culturally appropriateness of survey items in the Ugandan setting, we conducted a two-stage pilot test. First, we administered the survey to study staff who were caregivers of children aged 6–48 months. Following the feedback received during the first pilot, we revised the survey, and then the refined survey was administered to caregivers of non-study children aged 6–48 months who visited Mulago Hospital. After the second pilot test, we revised survey items again and created the final 94-item version to be used for data collection. Other variables of interest collected in the same survey were demographic characteristics (gender, age, marital status, education, religion, phone ownership, number of children within the household). We translated the final versions of the survey into Luganda and Lusoga, the local languages of Kampala and Jinja, respectively. The survey was imported into REDCap to enable mobile survey entry. Surveys were conducted by trained interviewers using the appropriate language depending on the participant’s preference.

Using the survey responses, we characterized caregiver’s Covid-19 related experiences and perceptions across nine domains: 1) pregnancy or birth-related stressors during COVID-19; 2) exposure and vulnerability to contacting COVID-19; 3) changes in living situations and physical activity during COVID-19; 4) perceived risk of COVID-19; 5) economic consequences of COVID-19; 6) social support during COVID-19; 7) food insecurity during COVID-19; 8) domestic violence during COVID-19; and 9) disruptions in healthcare access and school/daycare. We allocated the same weights (maximum of one) for each item—detailed information of items under each section are presented in the **S1 Table**. We generated the sub-total score for each of the nine sections by summing the scores of individual items within each section. To ensure each section had equal weight regardless of the number of items, we standardized the sub-total scores to a mean of 0 and a standard deviation of 1. Then, we created the total score by summing up the nine standardized section scores. A higher section or total score indicates worse COVID-19-related experiences and perceptions.

#### HSCL-25

Caregiver depression and anxiety were assessed using *the Hopkins Symptom Checklist (HSCL-25)* [28]. The HSCL-25 uses 25 items and asks participants to respond on a four-point Likert scale how frequently they experienced depression (15 items) and anxiety-related symptoms (10 items) during the last week: not at all, a little, quite a bit, and extremely. The total possible score ranges from 0 to 60 with higher HSCL-25 scores indicating greater depression and anxiety [29]. Mean scores for anxiety and depression subscales were calculated after dividing the total HSCL-25 score by the number of items [30]. An HSCL-25 score with a mean of 1.75 or greater is considered clinically significant to discern the presence of depression and anxiety [30]. HSCL-25 has been widely used and validated in East African populations including Uganda [30–32]. In the current study, the HSCL-25 showed high reliability with a Cronbach’s alpha of .91. Another Ugandan study also reported high reliability of the HSCL-25 (Cronbach’s alpha = .89) [33].

#### CESD-20

Caregiver depression was measured by *the Center for Epidemiologic Studies Depression (CESD-20) scale* [34]. The CESD-20 is a widely used survey with 20 items (total possible score ranging from 0 to 60), screening for the presence of significant depressive symptoms [34]. CESD-20 asks participants to report, on a four-point scale (0 = rarely/none of the time to 3 = all the time), the frequency of symptoms for 20 scale assessment items [34]. Higher CESD-20 scores indicate higher perceived depressive symptoms [34]. We used CESD-20 along with HSCL-25 to have a more comprehensive assessment of the caregivers’ mental health and to enable cross-validation of the study findings. The full 20-item CESD-20 has been validated to screen depression in different sub-Saharan African countries including Uganda [35–37]. The current study tested the reliability of CESD-20 and confirmed the high reliability with Cronbach’s alpha of .88 in the study population. Previous study based in Northern Uganda confirmed the high internal consistency (Cronbach’s alpha = .92) of CESD-20 in its study population [35]. The same study also reported high predictive validity of CESD-20 by comparing CESD scores with participants’ Mini-International Neuropsychiatric Interview scores, a clinician administered tool used to diagnose depression [35]. A total CESD-20 score of 16 or greater is considered as indicative of depressive symptoms in the general population and has been validated its use as a cut-off point to assess depression in Ugandan studies [34, 38, 39].

#### Questionnaire of material possessions

Information on socioeconomic status (SES) was collected by trained staff through a home visit which took place one week after enrollment. SES was obtained using a questionnaire of material possessions assessing housing quality (type of roof, water supply, and cooking fuel) and the presence of key items (e.g., electricity, shoes for subject, radio, television, bicycle, motorcycle, motor vehicle, and animals). Each item was weighted and summed up to a total score with a potential range of 0 to 27. The total SES score was used as a proxy measure of household standards of living by summing up commonly found household assets, which is a commonly used measure in Uganda [40, 41].

### Ethics statement

All caregivers gave informed written consent for their participation in the COVID substudy. The consent process was administered in Luganda, Lusoga, or English, according to the caregiver’s preference. If a caregiver was unable to read or write, he or she indicated consent with a thumbprint, and a witness who was present at the entire consent process, also signed the consent form. This study was approved by the University of Minnesota IRB and the Mulago Hospital Research Ethics Committee.

### Inclusivity in global research

This study has been approved by the Institutional Review Boards (IRBs) and Research Ethics Committees (RECs) of both Mulago Hospital and the University of Minnesota. It has also received approval from the National Drug Authority (NDA) and the Uganda National Council for Science and Technology (UNCST). Additional information regarding the ethical, cultural, and scientific considerations specific to inclusivity in global research is included in the Supporting Information (**S1 Checklist**).

### Statistical analysis

Bivariate analysis (Wilcoxon rank sum test for continuous predictors and Fisher’s exact test for categorical predictors) was employed to assess the association between caregivers’ characteristics and COVID survey responses with the presence of depressive symptoms (CESD-20 ≥ 16) and/or anxiety (mean HSCL-25 ≥ 1.75). Separate multiple linear regression (MLR) models were used to assess the relationship between each of COVID-19 survey scores (each of the nine COVID-19 survey section scores and COVID-19 survey total score; treated as predictors) and symptoms of depression and/or anxiety (HSCL-25 and CESD-20 treated as continuous; treated as outcomes). We analyzed adjusted models controlled for caregiver’s age, education level (never attended school, attended primary or secondary school, attended tertiary school or above), marital status (never in union, married or have a partner, divorced or separated), SES score, and child malaria status (having malaria, having no malaria). Covariates were selected based on their associations with the COVID-19 related stressors, anxiety, and depression from previous studies [20, 42, 43]. We confirmed that the outcomes (HSCL-25 or CESD-20 scores) were reasonably normally distributed and had no outliers, using the Q-Q plot, and no issues with homoscedasticity, based on the residual plots.

Child malaria status was included as a covariate, based on the observation that the associations between the COVID-19 scores and the HSCL-25 or CESD-20 scores did not vary by child malaria status which was confirmed by no significant interaction between COVID-19 scores and child malaria status for both outcomes (**S2 Table**). Additionally, we performed MLR stratified by child age to explore if there is a difference in association between COVID-19 experiences and mental health among caregivers who take care of infants or at early toddler period (younger than 18 months) versus whose children are at later toddler period (18 months or older).

The details of the sample size calculation for the current study are as follows: We based our sample size on the primary outcome of the study (e.g., HSCL-25 score) at enrollment or the Month 12 study visit. The required sample size was calculated using G*Power software version 3.1.9.4, based on a Pearson’s correlation effect size of .25 (alpha = .05, power = 80%). We included 100 caregivers, accounting for a 20% loss to follow-up. All statistical analyses were performed using STATA version 14.0 and R version 4.1.1.

## Results

**Table 1** shows the characteristics of the study population. Most primary caregivers were mothers of the study children (87%), with a mean (SD) age of 28 (8) years (**Table 1**). Over 90% of the caregivers had completed upper primary school, and approximately 80% were married or had a partner. Nearly half of the caregivers had a mean HSCL-25 score ≥ 1.75 (49%) and a total CESD-20 score ≥ 16 (46%), indicative of having symptoms of depression and/or anxiety.

**Table 1:** Sociodemographic characteristics of primary caregivers of young children^1^.

Indicator	Mean ± SD or N (%)
**N**	100
**Age, y; mean (SD)**	28.3 (7.8)
**Mother to child, n (%)**	87 (87.0)
**Education level, n (%)**	
Never attended	4 (4.0)
Completed primary or secondary	88 (88.0)
Completed tertiary or above	8 (8.0)
**Marital status, n (%)**	
Never in union	7 (7.0)
Married or have a partner	82 (82.0)
Divorced or separated	11 (11.0)
**Number of children, mean (SD)**	2.5 (1.7)
**Socioeconomic status (SES) score, mean (SD)**	13.2 (3.1)
**Child had malaria at enrollment, n (%)**	85 (85.0)
**Caregiver hemoglobin (g/dL), mean (SD)**	13.3 (1.4)
**Total HSCL-25 score, mean (SD)**	45.5 (13.5)
**Mean of HSCL-25 ≥1.75, n (%)**	49 (49.0)
**Total CESD-20 score, mean (SD)**	15.8 (10.9)
**CESD-20≥16, n (%)**	46 (46.0)

HSCL, Hopkins Symptom Checklist; CESD, Center for Epidemiologic Studies Depression; SES, socioeconomic status.

^1^Characteristics of 100 Ugandan caregivers of young children living in an urban slum near Kampala and Jinja who took part in a cross-sectional survey of COVID-19 related experiences and perceptions from October 2022 to April 2023. Values are mean (SD), or n (%).

**Table 2** shows the results of the Wilcoxon rank-sum test for continuous variables and Fisher’s exact test for categorical variables, examining differences in caregiver characteristics based on their mental health status (with or without clinically significant depression and/or anxiety). None of the characteristics considered (age, relationship to child, education level, marital status, number of children within the household, phone ownership, hemoglobin level, socioeconomic score, or child having malaria) were significantly associated with symptoms of depression and/or anxiety. MLR models that included the same set of caregivers’ characteristics as predictors and HSCL-25 or CESD-20 as a continuous outcome also found no characteristics associated with depression and/or anxiety (**S3 Table**).

**Table 2:** Caregivers’ baseline characteristics by their depression and/or anxiety status^1^.

Variable	HSCL-2 (anxiety and depression)			CESD-20 (depression)		
	Mean HSCL-25 < 1.75 (N = 51)	Mean HSCL-25 ≥ 1.75 (N = 49)	p-value^2^	CESD-20<16 (N = 54)	CESD-20≥16 (N = 46)	p-value^2^
**Age**	28.2 (8.1)	28.3 (7.7)	> .99	28.9 (8.3)	27.4 (7.3)	.35
**Relationship to children**			.55			.37
Mother	43 (84.3%)	44 (89.8%)		45 (83.3%)	42 (91.3%)	
Other	8 (15.7%)	5 (10.2%)		9 (16.7%)	4 (8.7%)	
**Education level**			.80			.10
Never attended	2 (3.9%)	2 (4.1%)		3 (5.6%)	1 (2.2%)	
Primary or secondary	46 (90.2%)	42 (85.7%)		44 (81.5%)	44 (95.7%)	
Tertiary or above	3 (5.9%)	5 (10.2%)		7 (13.0%)	1 (2.2%)	
**Marital status**			.29			.22
Never in union	4 (7.8%)	3 (6.1%)		6 (11.1%)	1 (2.2%)	
Married or have a partner	39 (76.5%)	43 (87.8%)		43 (79.6%)	39 (84.8%)	
Divorced or separated	8 (15.7%)	3 (6.1%)		5 (9.3%)	6 (13.0%)	
**Number of children**	2.1 (.9)	2.1 (.8)	.99	2.1 (.8)	2.0 (.8)	.86
**Own phone**			.39			.56
No	9 (17.6%)	5 (10.2%)		9 (16.7%)	5 (10.9%)	
Yes	42 (82.4%)	44 (89.8%)		45 (83.3%)	41 (89.1%)	
**Hemoglobin level (g/dL)**	13.3 (1.4)	13.3 (1.5)	.78	13.3 (1.5)	13.3 (1.3)	.80
**SES score, mean (SD)**	13.3 (3.2)	13.2 (3.0)	.93	13.3 (3.4)	13.1 (2.8)	.88
**Child having malaria**			.26			.40
No	10 (19.6%)	5 (10.2%)		10 (18.5%)	5 (10.9%)	
Yes	41 (80.4%)	44 (89.8%)		44 (81.5%)	41 (89.1%)	

HSCL, Hopkins Symptom Checklist; CESD, Center for Epidemiologic Studies Depression; SES, socioeconomic status.

^1^Median (IQR); N (%).

^2^P-values were calculated using Wilcoxon rank sum test for categorical variables and using Fisher’s exact tests for continuous variables.

**Table 3** reports the caregivers’ responses to each COVID-19 survey question by the presence of anxiety and/or depressive symptoms measured by HSCL-25 and CESD-20, using Fisher’s exact test. The proportion of caregivers who gave birth during the COVID-19 lockdowns (Q1-3) was significantly higher among those with depressive symptoms compared to those without depressive symptoms (CESD-20 score ≥ 16 vs. < 16; 67% vs. 46%, p = .04). Caregivers with depressive symptoms were also more likely to report a decrease in physical activity during the first COVID-19 total lockdowns (Q3-2) (CESD-20 score ≥ 16 vs. < 16; 83% vs. 56%, p = .01). Experiencing a whole day and night without eating (Q7-3) was significantly more prevalent among caregivers with symptoms of depression and/or anxiety compared to those without these symptoms (mean HSCL-25 score ≥ 1.75 vs. < 1.75; 49% vs. 22%, p = .001, CESD-20 score ≥ 16 vs. < 16; 48% vs. 24%, p = .02). The likelihood of witnessing physical violence towards a child by an adult in the household (Q8-1) was higher among caregivers with depressive symptoms (CESD-20 score ≥ 16 vs. < 16; 15% vs. 2%, p = .02). Additionally, participants with depressive symptoms were more likely to report an increase in childcare needs since the start of COVID-19 (Q9-3) (CESD-20 score ≥ 16 vs. < 16; 26% vs. 9.3%, p = .03).

**Table 3:** Results of Fisher’s exact test [N (%)] for test of association between caregivers’ responses to COVID-19 survey and depression and/or anxiety status.

	HSCL-25 (symptoms of anxiety and depression)			CESD-20 (depressive symptoms)		
Factor	Mean HSCL-25 < 1.75	Mean HSCL-25 ≥ 1.75	p-value	CESD-20 < 16	CESD-20 ≥ 16	p-value
**Section 1: Pregnancy or birth-related stressors during COVID-19**						
Q1-1. Are you currently pregnant?			> .99			> .99
*No*	50 (98%)	48 (98%)		53 (98%)	45 (98%)	
*Yes*	1 (2%)	1 (2%)		1 (2%)	1 (2%)	
Q1-2. Have you given birth before the COVID-19 outbreak (Oct 2019 to March 30, 2020)?			.76			> .99
*No*	46 (90%)	43 (88%)		48 (89%)	41 (89%)	
*Yes*	5 (10%)	6 (12%)		6 (11%)	5 (11%)	
Q1-3. Have you given birth during the COVID-19 lockdowns (March 31, 2020 to January, 2022)?			.16			.04
*No*	26 (51%)	18 (37%)		29 (54%)	15 (33%)	
*Yes*	25 (49%)	31 (63%)		25 (46%)	31 (67%)	
Q1-4. Have you given birth after the COVID-19 lockdowns (February 2022)?			.11			.79
*No*	39 (76%)	44 (90%)		44 (81%)	39 (85%)	
*Yes*	12 (24%)	5 (10%)		10 (19%)	7 (15%)	
Q1-5. How worried have you been that you might get COVID-19 while pregnant?			.36			.07
*Not worried at all or NA*	20 (39%)	16 (33%)		25 (46%)	11 (24%)	
*Somewhat worried*	11 (22%)	17 (35%)		12 (22%)	16 (35%)	
*Very worried*	20 (39%)	16 (33%)		17 (31%)	19 (41%)	
Q1-6. How worried have you been that your baby (born just before, during, or after Covid-19 lockdowns) might get COVID-19?			.82			.14
*Not worried at all or NA*	20 (39%)	19 (39%)		26 (48%)	13 (28%)	
*Somewhat worried*	11 (22%)	13 (27%)		11 (20%)	13 (28%)	
*Very worried*	20 (39%)	17 (35%)		17 (31%)	20 (43%)	
Q1-7. Has COVID-19 negatively impacted your expectations about your pregnancy/birth?			.15			.16
*Not worried at all or NA*	26 (51%)	21 (43%)		29 (54%)	18 (39%)	
*Somewhat worried*	17 (33%)	12 (24%)		16 (30%)	13 (28%)	
*Very worried*	8 (16%)	16 (33%)		9 (17%)	15 (33%)	
**Section 2: Exposure and vulnerability to contacting COVID-19**						
Q2-1. Since the start of the outbreak, have you have symptoms of COVID-19?			.32			.08
*No*	41 (84%)	37 (76%)		47 (87%)	33 (72%)	
*Yes*	8 (16%)	12 (24%)		7 (13%)	13 (28%)	
Q2-2. During the COVID-19 outbreak, have you ever had a close contact with someone that was sick with COVID- 19?			> .99			.18
*No*	42 (82%)	41 (84%)		42 (78%)	41 (89%)	
*Yes*	9 (18%)	8 (16%)		12 (22%)	5 (11%)	
Q2-3. Did any of them die because of COVID-19?			> .99			>.99
*No or NA*	47 (92%)	46 (94%)		50 (93%)	43 (93%)	
*Yes*	4 (8%)	3 (6%)		4 (7%)	3 (7%)	
**Section 3: Changes in living situations and physical activity during COVID-19 (Social isolation)**						
Q3-1. [Was there/Has there been] a change in where you live or who you live(d) with since the COVID-19 outbreak?			.68			.01
*No*	33 (65%)	29 (59%)		34 (63%)	28 (61%)	
*Yes*	18 (35%)	20 (41%)		20 (37%)	18 (39%)	
Q3-2. Decrease in physical activity during the first lockdowns first COVID-19 total lockdowns (March—June 2020)			.29			.01
*No*	19 (37%)	13 (27%)		24 (44%)	8 (17%)	
*Yes*	32 (63%)	36 (73%)		30 (56%)	38 (83%)	
Q3-3. Decrease in physical activity during the second COVID-19 total lockdowns (June—July 2021)			>.99			.55
*No*	25 (49%)	25 (51%)		29 (54%)	21 (46%)	
*Yes*	26 (51%)	24 (49%)		25 (46%)	25 (54%)	
**Section 4: Perceived risk of COVID-19**						
Q4-1. How concerned are you about the spread of COVID-19 in your community?			.65			.93
*Not concerned*	31 (61%)	27 (55%)		32 (59%)	26 (57%)	
*Somewhat concerned*	18 (35%)	21 (43%)		20 (37%)	19 (41%)	
*Very concerned*	2 (4%)	1 (2%)		2 (4%)	1 (2%)	
Q4-2. Approximately how many people in your community do you think are or have been infected with Covid-19?			.04			.09
*No one has been infected*	24 (47%)	12 (24%)		22 (41%)	14 (30%)	
*Some people*	26 (51%)	34 (69%)		31 (57%)	29 (63%)	
*Most people*	1 (2%)	3 (6%)		1 (2%)	3 (7%)	
Q4-3. How concerned are you about getting infected yourself?			.95			.49
*Not concerned*	31 (61%)	28 (57%)		31 (57%)	28 (61%)	
*Somewhat concerned*	17 (33%)	18 (37%)		21 (39%)	14 (30%)	
*Very concerned*	3 (6%)	3 (6%)		2 (4%)	4 (9%)	
**Section 5: Economic consequences of COVID-19**						
Q5-1. During/after the first total lockdowns (March–June 2020), has your job or source of income been affected by Covid-19?			.55			.69
*No or NA*	23 (45%)	26 (53%)		25 (46%)	24 (52%)	
*Yes*	28 (55%)	23 (47%)		29 (54%)	22 (48%)	
Q5-2. During/after the second total lockdowns (June—July 2021), has your job or source of income been affected by Covid-19?			.41			.68
*No or NA*	31 (61%)	34 (69%)		34 (63%)	31 (67%)	
*Yes*	20 (39%)	15 (31%)		20 (37%)	15 (33%)	
Q5-3. After the Coronavirus (COVID-19) lockdowns, how much of a loss of income has your household experienced?			.04			.03
*None*	6 (12%)	7 (14%)		7 (13%)	6 (13%)	
*Complete*	25 (49%)	12 (24%)		26 (48%)	11 (24%)	
*Partial*	20 (39%)	30 (61%)		21 (39%)	29 (63%)	
Q5-4. Are you worried about the impact of Covid-19 on your household’s finances in the future?			> .99			.79
*No*	8 (16%)	8 (16%)		8 (15%)	8 (17%)	
*Yes*	43 (84%)	41 (84%)		46 (85%)	38 (83%)	
Q5-5. Are you more economically reliant on your husband/partner now than before the Covid-19 outbreak (March 2020)?			.31			.11
*No or NA*	33 (65%)	26 (53%)		36 (67%)	23 (50%)	
*Yes*	18 (35%)	23 (47%)		18 (33%)	23 (50%)	
**Section 6: Absence of social support during COVID-19**						
Q6-1. Do you think you can count on your family members or friends for financial assistant when you need it?			.68			.83
*No*	32 (63%)	33 (67%)		36 (67%)	29 (63%)	
*Yes*	19 (37%)	16 (33%)		18 (33%)	17 (37%)	
Q6-2. Do you feel that if you needed non-material help (e.g. somebody to talk to, help with childcare) you could receive it from relatives, friends, neighbors or other persons that you know?			.23			.34
*No*	37 (73%)	41 (84%)		40 (74%)	38 (83%)	
*Yes*	14 (27%)	8 (16%)		14 (26%)	8 (17%)	
**Section 7: Food insecurity during COVID-19**						
Q7-1. Since the Coronavirus (COVID-19) began, would you say that you have eaten your favorite food less?			.33			.47
*No*	13 (25%)	8 (16%)		13 (24%)	8 (17%)	
*Yes*	38 (75%)	41 (84%)		41 (76%)	38 (83%)	
Q7-2. Since the Coronavirus (COVID-19) began, has the number of meals or times you eat in a day reduced?			.22			.54
*No*	23 (45%)	16 (33%)		23 (43%)	16 (35%)	
*Yes*	28 (55%)	33 (67%)		31 (57%)	30 (65%)	
Q7-3. Since the Coronavirus (COVID-19) began, is “going a whole day and night without eating anything” more common for you or your household member now compared to before Covid-19?			.001			.02
*No*	40 (78%)	25 (51%)		41 (76%)	24 (52%)	
*Yes*	11 (22%)	24 (49%)		13 (24%)	22 (48%)	
**Section 8: Domestic violence during COVID-19**						
Q8-1. Since the Coronavirus (COVID-19) began, has the event where an adult you were living with got physically violent with a child (for example, shoved, hit, kicked, or shook [her/him/them]) happened more frequently?			.16			.02
*No or NA (never happened)*	49 (96%)	43 (88%)		53 (98%)	39 (85%)	
*Yes*	2 (4%)	6 (12%)		1 (2%)	7 (15%)	
Q8-2. Since the Coronavirus (COVID-19) began, has the event where an adult in your household was physically violent with you (for example, shoved, hit, kicked, or shook you) happened more frequently?			.36			.62
*No or NA (never happened)*	50 (98%)	46 (94%)		51 (94%)	45 (98%)	
*Yes*	1 (2%)	3 (6%)		3 (6%)	1 (2%)	
Q8-3. Since the Coronavirus (COVID-19) began, has the event where an adult in your household was emotionally violent with you happened more frequently?			.06			.29
*No or NA (never happened)*	46 (90%)	37 (76%)		47 (87%)	36 (78%)	
*Yes*	5 (10%)	12 (24%)		7 (13%)	10 (22%)	
**Section 9: Disruptions in healthcare access and school/daycare**						
Q9-1. Have you experienced any difficulties accessing healthcare services since the Coronavirus (COVID-19) began?			.42			.55
*No*	29 (57%)	23 (47%)		30 (56%)	22 (48%)	
*Yes*	22 (43%)	26 (53%)		24 (44%)	24 (52%)	
Q9-2. Since the Coronavirus (COVID-19) began, has your child’s school or daycare been closed for any length of time as a result of coronavirus, or not?			.25			.06
*No or NA (too young to go to school or daycare)*	41 (80%)	34 (69%)		45 (83%)	30 (65%)	
*Yes*	10 (20%)	15 (31%)		9 (17%)	16 (35%)	
Q9-3. Since the Coronavirus (COVID-19) began, have you experienced increases in the childcare needs compared to before COVID-19?			.79			.03
*No*	43 (84%)	40 (82%)		49 (91%)	34 (74%)	
*Yes*	8 (16%)	9 (18%)		5 (9.3%)	12 (26%)	

**Table 4** presents the results from the MLR between the COVID-19 survey scores (nine section scores and the total score) and continuous HSCL-25 and CESD-20 scores. Each section represents different features of COVID-19-related experiences among caregivers. Food insecurity during COVID-19 (Section 7) and the total COVID-19 survey score were significantly associated with both HSCL-25 and CESD-20 scores. A 1-unit higher food insecurity during COVID-19 score was associated with a 3.25-point higher HSCL-25 score (95% CI [0.41, 6.10], p = .03) and a 3.09-point higher CESD-20 score (95% CI [0.79, 5.39], p = .01). Child age-stratified (< 18 months vs. ≥ 18 months) MLR results (**S4 Table**) showed that the association between food insecurity during COVID-19 score and caregiver’s HSCL-25 and/or CESD-20 score significantly varied by the child age group (p-interaction = .02 for both HCSL-25 and CESD-20). Only among caregivers of children younger than 18 months (N = 42), higher food insecurity during COVID-19 score was significantly associated with higher HSCL-25 and CESD-20 scores (HSCL-25: β = 7.30, 95% CI [3.27, 11.33], CESD-25: β = 6.65, 95% CI [3.35, 9.96]).

**Table 4:** Multiple linear regression results between caregivers’ COVID-19 related experience and perceptions and their HSCL-25 or CESD-20 scores (N = 100).

		HSCL-25 (depression and anxiety)^2^		CESD-20 (depression)^2^	
Predictor^1^		Estimate (95% CI)^3^	p-value	Estimate (95% CI)^3^	p-value
Section 1	Pregnancy or birth-related stressors during Covid-19	1.82 (-1.27, 4.91)	.25	2.19 (-0.31, 4.68)	.09
Section 2	Vulnerability to contacting Covid-19	0.37 (-2.40, 3.15)	.79	-0.62 (-2.89, 1.64)	.59
Section 3	Change in living situations and physical activity during Covid-19	2.33 (-0.63, 5.30)	.13	3.35 (1.00, 5.70)	.01
Section 4	Perceived risk of COVID-19	2.33 (-0.49, 5.16)	.11	1.89 (-0.42, 4.19)	.11
Section 5	Economic consequences of COVID-19	3.00 (0.15, 6.34)	.07	2.11 (-0.53, 4.74)	.12
Section 6	Absence of social support during COVID-19	-0.87 (-3.63, 1.89)	.54	-0.74 (-3.00, 1.51)	.52
Section 7	food insecurity during COVID-19	3.25 (0.41, 6.10)	.03	3.09 (0.79, 5.39)	.01
Section 8	Domestic violence during COVID-19	3.82 (0.94, 6.70)	.01	2.29 (-0.11, 4.68)	.06
Section 9	Disruptions in healthcare access and school/daycare	1.69 (-1.15, 4.54)	.25	2.24 (-0.05, 4.53)	.06
Total score	Summation of 9 section scores	1.44 (0.64, 2.25)	< .001	1.28 (0.63, 1.93)	< .001

HSCL, Hopkins Symptom Checklist; CESD, Center for Epidemiologic Studies Depression.

^1^All section scores were standardized (Mean = 0, SD = 1)

^2^Models were adjusted for caregiver’s age, education level, marital status, SES score, and child having malaria.

^3^Increase in HSCL or CESD-20 score per 1 SD increase in each section score.

A 1-unit higher score in the total COVID-19 survey score was associated with a 1.44-point higher HSCL-25 score (95% CI [0.64, 2.25], p < .001) and a 1.28-point higher CESD-20 score (95% CI [0.63, 1.93], p < .001). Additionally, a 1-unit higher score in domestic violence during COVID-19 section (Section 8) score was associated with a 3.82-point higher HSCL-25 score (95% CI [0.94, 6.70], p = .01). A 1-unit higher Section 3 score, which indicates greater changes in household members, frequency of movement, or reductions in physical activity during COVID-19, was associated with a 3.35-point higher CESD-20 score (95% CI [1.00, 5.70], p = .01). Adjusted R^2^ values for all associations are indicated in **S5 Table**.

## Discussion

Our study aimed to examine the cross-sectional associations between COVID-19-related perceptions and experiences and symptoms of depression and/or anxiety among 100 Ugandan caregivers of young children, including those with malaria and iron deficiency (N = 85) and those without malaria or anemia (N = 15). We found that approximately half of the caregivers had clinically significant symptoms of depression and anxiety. Adverse experiences, including changes in living situations (e.g., movement or changes in household members) and reduced physical activity, food insecurity, and domestic violence during the COVID-19 period, were associated with more severe signs of depression and/or anxiety. These associations did not vary based on whether the caregivers were caring for children with malaria.

Our study found that close to half of the Ugandan caregivers enrolled in this study had clinical symptoms of depression and/or anxiety. Although studied in different groups using different tools, other recent studies in Uganda also reported high levels of mental health problems due to COVID-19 pandemic [13–15, 19]. A mobile phone-based cross-sectional survey conducted between the first and second total lockdowns (December 2020 to April 2021) reported that nearly half of the respondents, adults older than 18 years, showed symptoms of moderate or severe mental distress as measured by the Patient Health Questionnaire (PHQ-4) [14]. Another online survey of university students in Uganda suggested that the majority of students experienced symptoms of depression, anxiety, and stress, as measured by the Depression Anxiety and Stress Scale (DASS-21) during the COVID-19 lockdowns [15]. However, it is important to note that this study, along with other indicated Ugandan studies, is cross-sectional in nature. Therefore, it is not possible to establish a causal relationship between COVID-19 and elevated levels of depression and anxiety among different populations in Uganda.

The high levels of depression and anxiety among mothers are concerning because it is highly likely that they will not receive proper care, given the limited capacity of mental healthcare systems in Uganda. The mental health care system in Uganda, like many other low- and middle- income countries, is significantly under-resourced. Nationally, there are only 53 psychiatrists (approximately one psychiatrist per million population), and most of them are located in urban centers, whereas 83% of the Ugandan population lives in rural areas [19]. Only 1% of the healthcare budget is allocated to mental health, and the available financial and human resources for mental care services are limited to major mental disorders such as bipolar disorders and schizophrenia [44]. Consequently, common mental health problems such as depression, trauma, and anxiety are left neglected and untreated [18]. In the cultural context of Uganda, the key mental support system is through strong social bonding and gatherings, with participation in religious gatherings being the main coping mechanism for Ugandans to deal with challenges in their lives [11]. However, as any type of social gatherings were prohibited during lockdowns, the mental support systems were disrupted, causing difficulties in coping with stressors caused by COVID-19 [19]. It is vital to investigate the short- and long-term mental health impact of COVID-19 and its lockdown measures to plan for a better support system and reduce potential lingering effects [14]. Taking the lessons from COVID-19, policy makers in Uganda should be aware of the psychological impact of pandemics and disease outbreaks and increase their efforts to improve mental health care of vulnerable groups.

According to our study findings, caregivers had more severe depressive symptoms (higher CESD-25 score) if they experienced changes in living situations or decreases in physical activity and food insecurity during COVID-19. Also, caregivers tend to have more serious symptoms of depression and anxiety (higher HSCL-25 score) if they experienced more food insecurity and domestic violence during COVID-19. Overall, caregivers who had more negative perceptions of and worse experiences with COVID-19 tended to show more severe symptoms of depression and/or anxiety. It is important to note that our results are based on an exploratory analysis with a small sample size, and thus, some of these findings could be due to chance.

Mothers are the major caregivers in Uganda. Maternal depression, whether postpartum or later in the life of the child, has been considered as an important risk factor for children’s development [45]. Maternal depression, particularly in the postpartum period, has a negative effect on mother-infant bonding because depressive symptoms interfere with parenting behavior [46, 47]. Although less studied than maternal depression, maternal anxiety alone or in combination with depression may also increase the risks of poor mother-child interactions and adversely affect child developmental outcomes [48]. Children of mothers with high anxiety tend to have a lower regulation of emotions, poorer motor development, and significantly impaired concentration [49]. To ensure optimal development of children in Uganda, a better understanding of the mental health under the influence of public health crisis like COVID-19 is needed to adequately plan for culturally sensitive interventions and services [50].

This study has several limitations. Firstly, most caregivers (85%) in this study had children with malaria because the study enrolled caregivers based on their children’s participation in another larger study, which primarily focused on children with malaria. To account for differences among caregivers of children with and without malaria, we adjusted for the child malaria group in our regression models. We also confirmed that there was no interaction between child malaria status and caregivers’ COVID-19 scores on HSCL-25/CESD-20 scores, indicating that the association between caregivers’ COVID-19-related experiences and perceptions and their HSCL-25/CESD-20 scores did not vary by the child’s illness. However, because there were a much smaller number of caregivers of healthy children compared to those with children with iron deficiency and malaria, adjusting for child malaria status as a covariate or assessing the interaction between child malaria status and caregivers’ COVID-19 survey scores on caregivers’ mental health status may not have been sufficient to confirm that the child’s illness had no influence. Thus, our study findings are subject to limited generalizability in this regard.

Secondly, caregiver depression and anxiety were only measured with rapid and subjective self-reporting instruments (HSCL-25 and CESD-20), thereby possibly compromising the measurement accuracy of caregiver mental health and other related factors. In addition, our study findings may be susceptible to recall bias, given the time gap of a few months to years between the enforcement of lockdowns and administration of the survey on caregivers’ experiences and perceptions related to COVID-19.

Thirdly, we used an internally developed survey to capture caregivers’ experiences and perceptions of COVID-19. Although this survey is based on existing COVID-19 experience surveys developed by renowned institutions (COVEX by the Centers for Disease Control and Prevention and IPUMS-PMA COVID-19 survey by Johns Hopkins University), questions were adjusted for the Ugandan context. We tailored the survey questions to reflect the opinions of Ugandan caregivers and confirmed that the questions are relevant and meaningful in the Ugandan context through pilot surveys. However, it is not formally validated for capturing COVID-19 experiences in Uganda. Additionally, some sections include a smaller number of questions (e.g., Section 6, which covers the absence of social support during COVID-19, includes only two questions) compared to other sections. This may have introduced different weights for each section when computing the total score and affected the results. To address this, we standardized each section score so that all sections have equal weight. Furthermore, some questions ask about caregivers’ experiences in the early stages of the COVID-19 outbreak (e.g., Q3-2: Decrease in physical activity during the first COVID-19 total lockdown from March to June 2020) while others ask about their current experiences or perceptions (e.g., Q4-1: How concerned are you about the spread of COVID-19 in your community?). Including questions about experiences and perceptions from mixed time frames may have challenged caregivers to accurately recall their memories, which may have contaminated our results.

Fourthly, this study may be limited by uncontrolled confounding due to scarcity of information available such as caregivers’ childhood trauma or mental health history prior to COVID-19. However, based on the previous literature on a similar topic, there are no critical confounders such as caregiver’s marital status or socioeconomic status, that are not included in the study.

Lastly, because information on pre-pandemic measures of caregiver mental health is not available, disentangling the impact of COVID-19 related experiences and perceptions on caregiver mental health from other associated factors is challenging. Therefore, only limited interpretation of the association between COVID-19 experience and caregiver mental health is possible. Furthermore, as mentioned earlier, this is cross-sectional study, which limits our ability to establish causality between caregiver’s COVID-19 experiences and their symptoms of depression and anxiety.

However, it is worth noting that this is the first study that explores the potential mental health burden among caregivers from COVID-19 in Uganda. Findings from this study add to the previous evidence on the effects of the pandemic and subsequent lockdowns on mental health of the caregivers of young children living in low-income settings and call for longitudinal studies to examine the long-term impacts of COVID-19 on the mental well-being of populations at risk. Such research is crucial to better characterize the psychological burden from pandemics like COVID-19 and their lasting consequences, and effectively plan to prevent and support mental health challenges among the vulnerable populations.

## Supporting information

S1 Checklist Inclusivity in global research. (DOCX)

S1 Table COVID-19 survey items were used to compute scores of caregivers’ COVID-19 related experiences and perceptions. (DOCX)

S2 Table P-value for the interaction term between caregivers’ COVID-19 survey scores and child malaria status on caregivers’ HSCL-25 or CESD-20 score in the multiple linear regression models (N = 100). (DOCX)

S3 Table Linear regression results between caregiver’s characteristics and their HSCL-25 or CESD-20 scores (N = 100). (DOCX)

S4 Table Multiple linear regression results between caregivers’ COVID-19 survey Section 7 score and their HSCL-25 or CESD-20 scores stratified by child age group. (DOCX)

S5 Table Adjusted R^2^ for the Multiple linear regression models between caregivers’ COVID-19 related experience and perceptions and their HSCL-25 or CESD-20 scores (N = 100). (DOCX)

S1 Dataset Dataset used in the manuscript. (CSV)
