# Supplementary material for: Association of COVID-19-related perceptions and experiences with depression and anxiety in Ugandan caregivers of young children with malaria and iron deficiency: A cross-sectional study
Source: PLoS One. 2024 Dec 10;19(12):e0314409. doi: 10.1371/journal.pone.0314409 (PMC11630577; doi:10.1371/journal.pone.0314409)
Supplement: S1 Checklist — (DOCX) [file pone.0314409.s001.docx]

Inclusivity in global research

**Ethical considerations, permits and authorship**

*This section is applicable to all research types.*

Provide details as to who granted permissions and/or consent for the study to take place in the Methods section of your manuscript. This should include the names of **all** ethics boards, governmental organizations, community leaders or other bodies that provided approval for the study. If individuals provided approval refer to these people by their role or title but do not list their name(s).
If there were any deviations from the study protocol after approval was obtained please provide details of these changes in the Methods section of your manuscript.
Did this study involve local collaborators that are residents of the country where the research was conducted or members of the community studied? If you do not have any authors from said communities, please provide an explanation for this below.
Everyone listed as an author should meet PLOS’ criteria for authorship and all individuals who meet these criteria should be included in the author byline, rather than the acknowledgements. For further information please see the journal’s Authorship Policy.

Co-investogators and co-authors Dr. Mupere and Dr. Bangirana are Ugandan and faculty at Makerere University. The study staff responsible for enrollment, survey administration, mental health screenings, and data entry, including study coordinator Dr. Reagan Baluku, were all Ugandan.

Reported on page number: N/A

Reported on page number: 13

**Human subjects research (e.g. health research, medical research, cross-cultural psychology)**

Did you obtain written informed consent from a representative of the local community or region before the research took place? How did you establish who speaks for the community? Details of written informed consent obtained from study participants should be reported separately in the Methods section of your manuscript.

Our study design was reviewed and revised based on feedback from the Ugandan Principal Investigator, who is a co-author of this study. The anticipated outcomes were chosen reflecting the advice from a Ugandan neuropsychologist at Mulago Hospital, who is also a co-author of this study. Additionally, the COVID-19 survey was tailored based on input from Ugandan caregivers (outside of our study participants) through two rounds of pilot testing.

We obtained written informed consent from all study participants through trained Ugandan study nurses or neuropsych testers using local languages (Luganda or Lusoga) that are comfortable for the participants (Please see Ethics Statement of manuscript).

The consent form included information on the introduction, study purpose, study procedures, potential risks and benefits of study participation, reimbursements and compensations, and the voluntary nature and confidentiality of the study. All this information was verbally explained by the study nurses or testers responsible for the consent process and was also provided in the written consent form. We received the participant's signature on the consent form after sharing all this information.

How did members of the local community provide input on the aims of the research investigation, its methodology, and its anticipated outcome(s)?

When engaging with the local community, how did you ensure that the informed consent documents and other materials could be understood by local stakeholders?

We excluded adults who lacked the capacity to consent or had diminished capacity to consent, including but not limited to, those with acute medical conditions, psychiatric disorders, neurological disorders, developmental disorders, and behavioral disorders. This was to ensure all participants fully understood the study and could make an informed decision about their participation.

Also, onsent documents were available in English, Luganda, and Lusoga to meet the language needs of the participants. During enrollment, study staff provided both verbal explanations and written consent forms. These forms clearly outlined the study's purpose, procedures, surveys, potential risks and benefits, voluntary participation, and confidentiality.

Will the findings of the research be made available in an understandable format to stakeholders in the community where the study was conducted (e.g. via a presentation, summary report, copies of publications, etc.)? Please provide details of how this will be achieved.

Research findings were presented in an understandable form to all Ugandan study staff who worked on this study and to Ugandan health providers at Jinja and Mulago hospitals, where this study was based. Additionally, copies of the publication will be shared with the Ugandan study staff and health providers.

**Non-human subjects research using specimens/ animals collected as part of the study, or those housed in archival collections. Examples include archaeology, paleontology, botany and zoology.**

Did the permission you obtained from a local authority to perform the study include an agreement on access to outputs and benefit sharing? This may include procedures to enable fair distribution of the benefits and resources arising from the research performed. Please include any details of Prior Informed Consent and Benefit Sharing Agreements obtained. These may be required by field-specific regulations, for example the Convention on Biological Diversity (CBD) and the associated Nagoya Protocol.

N/A

If the material used in your study was imported, please A) provide the year it was imported and B) indicate whether permits were obtained to import/export the materials used, C) provide details of any permits obtained. If this information is not available, please indicate this.

N/A

If you used archival specimens, please state how the material used in your study was acquired by the institute it is held in and provide details of any permits obtained for the original excavations/ sample collection. If this information is not available, please indicate this.

N/A

How was the potential cultural significance of the materials collected in your study to local communities considered in your research design? Were Indigenous peoples and/or local researchers and institutions involved with archaeological excavations / collection of specimens? If so, please provide a description of their involvement.

N/A

If your manuscript includes photographs of human remains please indicate whether authors obtained permission from descendants or affiliated cultural communities to do so.

N/A
