# Supplementary material for: Association of COVID-19-related perceptions and experiences with depression and anxiety in Ugandan caregivers of young children with malaria and iron deficiency: A cross-sectional study
Source: PLoS One. 2024 Dec 10;19(12):e0314409. doi: 10.1371/journal.pone.0314409 (PMC11630577; doi:10.1371/journal.pone.0314409)
Supplement: S1 Table — (DOCX) [file pone.0314409.s002.docx]

**S1 Table.** COVID-19 survey items were used to compute scores of caregivers’ COVID-19 related experiences and perceptions.

| **Section 1: Pregnancy or birth-related stressors during COVID-19** | | |
| --- | --- | --- |
| Q1-1 | Are you currently pregnant? | 1=Yes 0=No or NA |
| Q1-2 | Have you given birth before the COVID-19 outbreak (Oct 2019 to March 30, 2020)? | 1=Yes 0=No or NA |
| Q1-3 | Have you given birth during the COVID-19 lockdowns (March 31, 2020 to January, 2022)? | 1=Yes 0=No or NA |
| Q1-4 | Have you given birth after the COVID-19 lockdowns (February 2022)? | 1=Yes 0=No or NA |
| Q1-5 | How worried have you been that you might get COVID-19 while pregnant? | 0= Not worried at all or NA 0.5= Somewhat worried 1= Very worried |
| Q1-6 | How worried have you been that your baby (born just before, during, or after Covid-19 lockdowns) might get COVID-19? | 0= Not worried at all or NA 0.5= Somewhat worried 1= Very worried |
| Q1-7 | Has COVID-19 negatively impacted your expectations about your pregnancy/birth? | 0= Not worried at all or NA 0.5= Somewhat worried 1= Very worried |
| **Section 2: Exposure and vulnerability to contacting COVID-19** | | |
| Q2-1 | Since the start of the outbreak, have you had symptoms of COVID-19? | 1=Yes 0=No |
| Q2-2 | During the COVID-19 outbreak, have you ever had a close contact with someone that was sick with COVID- 19? | 1=Yes 0=No |
| Q2-3 | Did any of them die because of COVID-19? | 1=Yes 0=No or NA |
| **Section 3: Changes in living situations and physical activity during COVID-19 (Social isolation)** | | |
| Q3-1 | [Was there/Has there been] a change in where you live or who you live(d) with since the COVID-19 outbreak? | 1=Yes 0=No |
| Q3-2 | Decrease in physical activity during the first COVID-19 total lockdowns (March - June 2020) | 1=Yes 0=No |
| Q3-3 | Decrease in physical activity during the second COVID-19 total lockdowns (June - July 2021) | 1=Yes 0=No |
| **Section 4: Perceived risk of COVID-19** | | |
| Q4-1 | How concerned are you about the spread of COVID-19 in your community? | 0=Not concerned 0.5=Somewhat concerned 1= Very concerned |
| Q4-2 | Approximately how many people in your community do you think are or have been infected with Covid-19? | 0=No one has been infected 0.5=Some people 1=Most people |
| Q4-3 | How concerned are you about getting infected yourself? | 0=Not concerned  0.5=Somewhat concerned 1= Very concerned |
| **Section 5: Economic consequences of COVID-19** | | |
| Q5-1 | During/after the first total lockdowns (March – June 2020), has your job or source of income been affected by Covid-19? | 1=Yes 0= No or NA |
| Q5-2 | During/after the second total lockdowns (June - July 2021), has your job or source of income been affected by Covid-19? | 1=Yes 0= No or NA |
| Q5-3 | After the Coronavirus (COVID-19) lockdowns, how much of a loss of income has your household experienced? | 0=None 0.5=Complete 1=Partial |
| Q5-4 | Are you worried about the impact of Covid-19 on your household’s finances in the future? | 1=Yes 0=No |
| Q5-5 | Are you more economically reliant on your husband/partner now than before the Covid-19 outbreak (March 2020)? | 1=Yes 0= No or NA |
| **Section 6: Absence of social support during COVID-19** | | |
| Q6-1 | Do you think you can count on your family members or friends for financial assistant when you need it? | 1=No 0=Yes |
| Q6-2 | Do you feel that if you needed non-material help (e.g. somebody to talk to, help with childcare) you could receive it from relatives, friends, neighbors or other persons that you know? | 1=No  0=Yes |
| **Section 7: Food insecurity during COVID-19** | | |
| Q7-1 | Since the Coronavirus (COVID-19) began, would you say that you have eaten your favorite food less? | 1=Yes 0=No |
| Q7-2 | Since the Coronavirus (COVID-19) began, has the number of meals or times you eat in a day reduced? | 1=Yes 0=No |
| Q7-3 | Since the Coronavirus (COVID-19) began, is “going a whole day and night without eating anything” more common for you or your household member now compared to before Covid-19? | 1=Yes 0=No |
| **Section 8: Domestic violence during COVID-19** | | |
| Q8-1 | Since the Coronavirus (COVID-19) began, has the event where an adult you were living with got physically violent with a child (for example, shoved, hit, kicked, or shook [her/him/them]) happened more frequently? | 1=Yes 0= No or NA (never happened) |
| Q8-2 | Since the Coronavirus (COVID-19) began, has the event where an adult in your household was physically violent with you (for example, shoved, hit, kicked, or shook you) happened more frequently? | 1=Yes 0= No or NA (never happened) |
| Q8-3 | Since the Coronavirus (COVID-19) began, has the event where an adult in your household was emotionally violent with you happened more frequently? | 1=Yes 0=No |
| **Section 9: Disruptions in healthcare access and school/daycare** | | |
| Q9-1 | Have you experienced any difficulties accessing healthcare services since the Coronavirus (COVID-19) began? | 1=Yes 0=No |
| Q9-2 | Since the Coronavirus (COVID-19) began, has your child’s school or daycare been closed for any length of time as a result of coronavirus, or not? | 1=Yes 0=No or NA (too young to go to school or daycare) |
| Q9-3 | Since the Coronavirus (COVID-19) began, have you experienced increases in the childcare needs compared to before COVID-19? | 1=Yes 0=No |
