# Supplementary material for: Association of COVID-19-related perceptions and experiences with depression and anxiety in Ugandan caregivers of young children with malaria and iron deficiency: A cross-sectional study
Source: PLoS One. 2024 Dec 10;19(12):e0314409. doi: 10.1371/journal.pone.0314409 (PMC11630577; doi:10.1371/journal.pone.0314409)
Supplement: S2 Table — (DOCX) [file pone.0314409.s003.docx]

**S2 Table.** P-value for the interaction term between caregivers’ COVID-19 survey scores and child malaria group on caregivers' HSCL-25 or CESD-20 score in the multiple linear regression models (N=100).

|  | **HSCL-25 (depression and anxiety)^3^** | **CESD-20 (depression)^3^** |
| --- | --- | --- |
| **Interaction term^1, 2^** | **p-interaction** | |
| **Section 1 score*child malaria group** | 0.18 | 0.12 |
| **Section 2 score*child malaria group** | 0.95 | 0.13 |
| **Section 3 score*child malaria group** | 0.96 | 0.05 |
| **Section 4 score*child malaria group** | 0.95 | 0.51 |
| **Section 5 score*child malaria group** | 0.98 | 0.18 |
| **Section 6 score*child malaria group** | 0.64 | 0.91 |
| **Section 7 score*child malaria group** | 0.48 | 0.95 |
| **Section 8 score*child malaria group** | 0.93 | 0.35 |
| **Section 9 score*child malaria group** | 0.80 | 0.34 |
| **Total score*child malaria group** | 0.34 | 0.64 |

HSCL, Hopkins Symptom Checklist; CESD, Center for Epidemiologic Studies Depression

^1^All scores were standardized (Mean=0, SD=1).

^2^Child malaria group indicates whether the caregiver's child had iron deficiency and malaria (primary children) or not (community children) at enrollment.

^3^Models were adjusted for caregiver's age, education level, marital status, and SES score.
