# Supplementary material for: Association of COVID-19-related perceptions and experiences with depression and anxiety in Ugandan caregivers of young children with malaria and iron deficiency: A cross-sectional study
Source: PLoS One. 2024 Dec 10;19(12):e0314409. doi: 10.1371/journal.pone.0314409 (PMC11630577; doi:10.1371/journal.pone.0314409)
Supplement: S3 Table — (DOCX) [file pone.0314409.s004.docx]

**S3 Table.** Linear regression results between caregiver’s characteristics and their HSCL-25 or CESD-20 scores (N=100).

|  | **HSCL-25 (symptoms of depression and anxiety)** | | **CESD (depressive symptoms)** | |
| --- | --- | --- | --- | --- |
|  | **Estimate (95% CI)** | **p-value** | **Estimate (95%)** | **p-value** |
| **Age** | 0.22 (-0.31 - 0.76) | 0.42 | -0.05 (-0.48 - 0.38) | 0.83 |
| **Relationship to children (ref: mother)** |  |  |  |  |
| Other | -2.86 (-13.24 - 7.52) | 0.59 | -3.46 (-11.74 - 4.83) | 0.42 |
| **Education level (ref: never attended)** |  |  |  |  |
| Primary or secondary | 3.95 (-10.57 - 18.47) | 0.60 | 4.64 (-6.96 - 16.24) | 0.43 |
| Tertiary or above | 0.03 (-17.36 - 17.42) | 1.00 | -1.92 (-15.81 - 11.96) | 0.79 |
| **Marital status (ref: never in union)** |  |  |  |  |
| Married or have a partner | 3.03 (-9.58 - 15.63) | 0.64 | 4.26 (-5.81 - 14.32) | 0.41 |
| Divorced or separated | -2.69 (-17.59 - 12.21) | 0.72 | 4.13 (-7.77 - 16.03) | 0.50 |
| **Number of children** | -0.37 (-4.65 - 3.91) | 0.87 | -0.12 (-3.54 - 3.30) | 0.95 |
| **Own phone (ref: no)** |  | 0.85 |  | 0.09 |
| Yes | 0.86 (-7.84 - 9.56) |  | 6.16 (-0.79 - 13.11) |  |
| **Hemoglobin level (g/dL)** | 0.28 (-1.89 - 2.44) | 0.80 | 0.80 (-0.93 - 2.53) | 0.37 |
| **Total SES score** | 0.11 (-0.83 - 1.05) | 0.82 | -0.07 (-0.82 - 0.68) | 0.86 |
| **Having children with malaria (ref: no)** |  | 0.15 |  | 0.30 |
| Yes | -5.92 (-13.94 - 2.10) |  | -3.42 (-9.83 - 2.99) |  |

HSCL, Hopkins Symptom Checklist; CESD, Center for Epidemiologic Studies Depression
