# Supplementary material for: Association of COVID-19-related perceptions and experiences with depression and anxiety in Ugandan caregivers of young children with malaria and iron deficiency: A cross-sectional study
Source: PLoS One. 2024 Dec 10;19(12):e0314409. doi: 10.1371/journal.pone.0314409 (PMC11630577; doi:10.1371/journal.pone.0314409)
Supplement: S4 Table — (DOCX) [file pone.0314409.s005.docx]

**S4 Table.** Multiple linear regression results between caregivers’ COVID-19 survey Section 7 score and their HSCL-25 or CESD-20 scores stratified by child age group.^1^

| **Predictor** | **Group** | **No. of caregivers** | **HSCL-25**  **(depression and anxiety)** | **CESD-20**  **(depression)** |
| --- | --- | --- | --- | --- |
|  |  |  | Coefficient (95% CI)3 | Coefficient (95% CI)3 |
| Section 7 score  (Food insecurity during COVID-19) | caregiver of older children (≥18 months) | 58 | 1.06 (-2.43, 4.55) | 1.16 (-1.70, 4.02) |
|  | caregiver of younger children (<18 months) | 42 | 7.30 (3.27, 11.33) | 6.65 (3.35, 9.96) |
|  | P-interaction^2^ |  | 0.02 | 0.02 |

HSCL, Hopkins Symptom Checklist; CESD, Center for Epidemiologic Studies Depression

^1^Models were adjusted for caregiver's age, education level, marital status, SES score, and child having malaria and included an interaction term between the Section 7 score and child age group (<18 months vs. ≥18 months).

^2^p-value for the interaction term between the Secion7 score and child age group.
