# Supplementary material for: Association of COVID-19-related perceptions and experiences with depression and anxiety in Ugandan caregivers of young children with malaria and iron deficiency: A cross-sectional study
Source: PLoS One. 2024 Dec 10;19(12):e0314409. doi: 10.1371/journal.pone.0314409 (PMC11630577; doi:10.1371/journal.pone.0314409)
Supplement: S5 Table — (DOCX) [file pone.0314409.s006.docx]

**S5 Table.** Adjusted R^2^ for the Multiple linear regression models between caregivers’ COVID-19 related experience and perceptions and their HSCL-25 or CESD-20 scores (N=100).^1^

|  | | **HSCL-25**  **(depression and anxiety)** | **CESD-20**  **(depression)** |
| --- | --- | --- | --- |
| **Predictor^2^** | | **Adjusted R^2^** | |
| **Section 1** | Pregnancy or birth-related stressors during Covid-19 | 0.00 | -0.01 |
| **Section 2** | Vulnerability to contacting Covid-19 | -0.02 | -0.04 |
| **Section 3** | Change in living situations and physical activity during Covid-19 | 0.07 | 0.08 |
| **Section 4** | Perceived risk of COVID-19 | 0.07 | 0.04 |
| **Section 5** | Economic consequences of COVID-19 | 0.10 | 0.06 |
| **Section 6** | Absence of social support during COVID-19 | 0.05 | 0.11 |
| **Section 7** | Food insecurity during COVID-19 | 0.09 | 0.02 |
| **Section 8** | Domestic violence during COVID-19 | 0.13 | 0.08 |
| **Section 9** | Disruptions in healthcare access and school/daycare | 0.07 | 0.04 |
| **Total score** | Summation of 9 section scores | 0.19 | 0.06 |

HSCL, Hopkins Symptom Checklist; CESD, Center for Epidemiologic Studies Depression.

^1^All Models were adjusted for caregiver's age, education level, marital status, SES score, and child having malaria.

^2^All section scores were standardized (Mean=0, SD=1)
